# Abnormality of Genital Organs

**Published:** 1895-03

**Authors:** 


ABNORMALITY OF GENITAL ORGANS —Prof. English (Vienna) reports an unusual anomaly of the genital organs. Patient presented a hypospadia, the canal being open almost the entire length of the pars pendula back to the scrotum. The glans and corpora cavernosa were completely divided to a point 2 c. m. behind the corona glandis; in erection the two parts became completely separated, coitus only being possible bv supporting them together by means of a condom. In this way the patient had become the father of one child. Patient was subjected to operation; the two parts of penis were united and a urethral canal constructed to the head of penis. Behind there remained still a small fistula. In literature there has been reported only one other such case.—*Wiener Klinische Rundschau.*

